# Starfruit-Induced Neurotoxicity Masked by Multiple Medical Confounders in a Hemodialysis Patient

**DOI:** 10.7759/cureus.112056

**Published:** 2026-07-04

**Authors:** Marcos Rosa Santana, Aura Flores, Rayan Alataa, Misbahuddin Khaja

**Affiliations:** 1 Department of Internal Medicine, BronxCare Health System, New York, USA

**Keywords:** end-stage renal disease (esrd), hemodialysis, seizure disorder, starfruit, starfruit-related encephalopathy, toxic encephalopathy

## Abstract

Starfruit (*Averrhoa carambola*) contains a potent neurotoxin, caramboxin, which can induce severe and often fatal encephalopathy in patients with impaired renal function. The diagnosis is frequently challenging due to nonspecific initial symptoms that can mimic other common complications in the end-stage renal disease (ESRD) population.

A 63-year-old female patient with ESRD on maintenance hemodialysis presented with intractable hiccups, emesis, and chest pain. Her hospital course was immediately complicated by a hypertensive emergency and an elevated troponin level. Other labs were significant for hyperkalemia (potassium 5.7 mEq/L) and high-anion-gap metabolic acidosis. Following a hemodialysis session, she developed a severe, fluctuating encephalopathy. In view of a sudden decline in mental status, the patient underwent a CT head and an MRI head, which were negative for acute findings. The diagnostic workup was confounded by multiple concurrent issues, including lumbar puncture results suggesting meningitis, seizure-like activity requiring antiepileptic drugs, and an electroencephalogram (EEG) suggestive of toxic-metabolic encephalopathy. Furthermore, the patient developed new-onset atrial fibrillation. On the fifth day of admission, a detailed over-the-counter and dietary history obtained from the family revealed a significant starfruit ingestion two days prior to symptom onset. With the diagnosis of starfruit intoxication established, management was refocused on intensive hemodialysis. The patient's mental status gradually improved with increased frequency of renal replacement therapy as the toxin was cleared and confounding medications were withdrawn.

This case illustrates how the classic presentation of starfruit neurotoxicity can be obscured by a complex clinical picture. It underscores that a high index of suspicion and a persistent, detailed dietary history are critical for a timely diagnosis and management of unexplained encephalopathy in all ESRD patients.

## Introduction

Starfruit (*Averrhoa carambola*) is a tropical fruit characterized by nutritional and medicinal benefits; however, it has also been linked to neurologic and nephrotic toxicity, due to breakdown of caramboxin (CBX), a potent neurotoxin, which can induce a severe and often fatal encephalopathy in patients with impaired renal function [1-3]. While individuals with normal kidney function efficiently clear this toxin, patients with end-stage renal disease (ESRD) are highly susceptible to its accumulation, even after consuming small quantities [4]. The clinical presentation of starfruit intoxication in uremic patients is characterized by a spectrum of neurological symptoms, mediated by an excitatory response, ranging from intractable hiccups and vomiting to more severe manifestations such as confusion, seizures, status epilepticus, and coma [5-7].

The diagnosis of starfruit intoxication is frequently delayed due to the nonspecific nature of the initial symptoms, which can mimic other common complications in the ESRD population, including uremic encephalopathy, electrolyte disturbances, or medication side effects. A high index of suspicion and a detailed dietary history are therefore paramount for timely diagnosis [8]. Management is centered on the prompt and efficient removal of CBX through intensive renal replacement therapy, as standard hemodialysis sessions may be insufficient [9]. Despite treatment, mortality rates remain high, underscoring the critical importance of patient education and prevention [6].

We present the case of a 63-year-old female patient on maintenance hemodialysis who developed severe, fluctuating encephalopathy after ingesting starfruit. The clinical picture was significantly complicated by multiple confounding factors, including a hypertensive emergency, suspected meningitis, and adverse effects from antiepileptic medications, which obscured the underlying diagnosis and posed considerable management challenges.

## Case presentation

A 63-year-old female patient with a medical history of ESRD on maintenance hemodialysis, hypertension, controlled type II diabetes mellitus, history of deep vein thrombosis not on anticoagulation, and bipolar disorder presented to the emergency department with a two-day history of chest wall pain, shortness of breath, inability to tolerate oral intake, emesis, and intractable hiccups.

On admission (Day 1), the patient was alert but exhibited intermittent periods of confusion. Her systolic blood pressure was in the 190s mmHg, consistent with a hypertensive emergency. The patient required ICU level of care, where the patient was started on a nicardipine infusion. Initial laboratory workup was notable for elevated troponins and several metabolic derangements (Table 1). An electrocardiogram showed sinus tachycardia without ischemic changes, and a chest X-ray was within normal limits. Nephrology was consulted for dialysis as per the patient's scheduled regimen.

**Table 1:** Laboratory values on admission

Laboratory test	Value	Reference range
Hemoglobin	10.7 g/dL	12.0-16.0 g/dL
Hematocrit	33.4%	42.0-51.0%
White blood cell	3.57 k/uL	4.8-10.8 k/uL
Platelets	200 k/uL	150-400 k/uL
Anion gap	21 mmol/L	9-15 mmol/L
Sodium	137 mEq/L	135-145 mEq/L
Potassium	5.7 mEq/L	3.5-5.0 mEq/L
Glucose	318 mg/dL	70-120 mg/dL
Blood urea nitrogen	48.0 mg/dL	6.0-20.0 mg/dL
Creatinine, serum	6.1 mg/dL	0.5-1.5 mg/dL
Troponin T, serum	86 ng/L	<12 ng/L

On hospital day 2, the patient underwent a scheduled hemodialysis session. Approximately 15 minutes into the treatment, she developed a severe, acute decline in mental status, becoming unable to follow commands. This prompted an urgent workup for encephalopathy. A CT scan of the head showed minimal periventricular white matter ischemic changes and opacification of the right mastoid cells, but no evidence of an acute intracranial hemorrhage or stroke. Given her persistent altered mental status with no clear focus of infection, a lumbar puncture was performed, which revealed an elevated cell count and protein/glucose with a neutrophilic predominance. On the recommendation of the Infectious Diseases team, she was started on broad-spectrum antimicrobial therapy (ceftriaxone, ampicillin, acyclovir, and metronidazole) for suspected meningitis and otomastoiditis.

Over hospital days 3 and 4, the patient's mental status continued to fluctuate, with periods of deep lethargy alternating with agitation. She was noted to have seizure-like episodes, and an initial electroencephalogram (EEG) was interpreted as showing toxic-metabolic and epileptic encephalopathy. The patient was then started on valproic acid. Concurrently, she developed new-onset atrial fibrillation with a rapid ventricular response and was started on apixaban (direct oral anticoagulant) for stroke prophylaxis. She continued to receive hemodialysis sessions, with only slight and transient improvements in her mental status.

On hospital day 5, a critical diagnostic clue emerged. Upon further, persistent questioning, the family disclosed that the patient had consumed multiple starfruits two days before her initial symptoms began. They had not mentioned it previously, as she had reportedly eaten a single starfruit in the preceding weeks with no recognized side effects. With this additional history, starfruit-induced neurotoxicity became the leading diagnosis.

Subsequent management was refocused on toxin removal through continued and intensive hemodialysis. The patient's antiepileptic regimen was reevaluated. A repeat EEG showed only mild encephalopathy with no definite epileptiform abnormalities. The valproic acid dose was decreased, and lacosamide was added; however, she became increasingly drowsy and difficult to arouse. On recommendation of the neurology team, all antiepileptic medications were discontinued, with minimal immediate improvement. Her course was further complicated by new bloody bowel movements, raising concern for a gastrointestinal bleed secondary to anticoagulation. Apixaban was held, and she required blood transfusions for anemia.

After multiple additional sessions of hemodialysis and cessation of the confounding medications, the patient's mental status slowly and steadily improved to her baseline, as confirmed by her family. The rectal bleeding resolved without further intervention. She completed a 14-day course of antibiotics as recommended by the infectious diseases team, and anticoagulation remained discontinued. The patient was safely discharged home with strict and comprehensive education to avoid any future consumption of starfruit, with routine hemodialysis reinstated.

## Discussion

This case illustrates a complex and challenging presentation of starfruit-induced neurotoxicity in a patient with ESRD. The patient's initial symptoms of hiccups, emesis, and subsequent rapid decline in mental status are classic features of this intoxication [6]. Nevertheless, the diagnosis was confounded by several concurrent medical issues, leading to a significant delay in identifying the etiology. This highlights the critical need for clinicians to maintain a high index of suspicion for starfruit toxicity in any ESRD patient presenting with unexplained neurological deterioration.

The pathophysiology of this condition is primarily driven by the neurotoxin CBX. CBX is a nonprotein amino acid with dual mechanisms: it acts as a potent agonist at glutamatergic N-methyl-D-aspartate receptors and inhibits gamma-aminobutyric acid receptors, leading to neuronal hyperexcitability, seizures, and neuronal death [2,5]. In ESRD, impaired renal clearance leads to its accumulation and passage across the blood-brain barrier, triggering severe encephalopathy [1,2]. The patient's presentation with confusion and seizure-like activity is a direct manifestation of this neurotoxic mechanism. Interestingly, the family reported a prior, smaller ingestion without side effects, which likely provided a false sense of security. This detail underscores that toxicity can be dose-dependent [7], and even a single previous uneventful exposure does not guarantee future safety. In contrast, in patients with normal renal function, CBX is generally effectively cleared; however, consumption of large quantities (e.g., large amounts of pure juice or multiple whole fruits on an empty stomach) can lead to acute oxalate nephropathy [10,11], mediated by oxalates contained in starfruit. In that sense, renal impairment ultimately reduces CBX clearance, resulting in neurotoxicity.

A crucial aspect of this patient’s case was the diagnostic delay. The diagnosis was only considered on the fifth day of admission following disclosure by the family. In the interim, the patient was extensively investigated for other causes of encephalopathy, including stroke, infection (meningitis), and metabolic derangements. The lumbar puncture results, which suggested meningitis, and the subsequent initiation of broad-spectrum antibiotics further complicated the picture. Moreover, the introduction and later withdrawal of antiepileptic medications for suspected seizures contributed to the patient's fluctuating mental status, making it difficult to discern the primary cause of her encephalopathy. This case powerfully argues for the inclusion of a detailed dietary history, specifically inquiring about exotic fruits such as starfruit, as a first-line investigation in any dialysis patient presenting with acute or subacute neurological changes.

The management of starfruit toxicity is yet to be defined, as standardized treatment remains limited. Early studies have elucidated that CBX is a water-soluble neurotoxin capable of being freely filtered through a 500 Da cutoff membrane, which supports the use of early hemodialysis or hemoperfusion for toxin removal, as CBX is a known dialyzable substance [9]. Nonetheless, standard intermittent hemodialysis is often insufficient to effectively clear the toxin load. The gradual improvement in our patient's mental status correlated with multiple ongoing hemodialysis sessions, consistent with the literature recommending intensive renal replacement therapy, such as daily or prolonged hemodialysis, continuous renal replacement therapy, or hemoperfusion [12]. It is also well-established that peritoneal dialysis is ineffective for CBX removal and should not be relied upon, particularly in patients with severe neurological symptoms [5]. Charcoal hemoperfusion has also been described as a potential alternative treatment for starfruit intoxication in patients with renal impairment, with increased extraction efficacy, rapid recovery of consciousness, lower ICU length of stay, and good clinical tolerance [13]. Moreover, N-acetylcysteine in rat models’ studies has been linked to protecting against starfruit-induced renal impairment through its antioxidant and anti-inflammatory properties, with implications of both nephrotoxicity and neurotoxicity [14].

## Conclusions

This case highlights starfruit intoxication should be considered in the differential diagnosis in patients with chronic kidney disease (CKD) or ESRD presenting with unexplained encephalopathy, seizures, or intractable hiccups. The diagnosis may be easily overlooked in a critically ill patient with multiple confounding factors, despite representing a potentially life-threatening toxicology emergency. A thorough history on the patient’s dietary habits is essential for early identification. Once identified, urgent and intensive hemodialysis be initiated for toxin clearance and improve clinical outcomes. Given the absence of definitive therapeutic guidelines, prevention remains paramount. Nephrologists, dietitians, and dialysis staff should consistently educate patients with CKD or ESRD to avoid the consumption of starfruit in any form.
